# Exposure to wood smoke increases arterial stiffness and decreases heart rate variability in humans

**DOI:** 10.1186/1743-8977-10-20

**Published:** 2013-06-06

**Authors:** Jon Unosson, Anders Blomberg, Thomas Sandström, Ala Muala, Christoffer Boman, Robin Nyström, Roger Westerholm, Nicholas L Mills, David E Newby, Jeremy P Langrish, Jenny A Bosson

**Affiliations:** 1 Department of Public Health and Clinical Medicine, Division of Medicine/Respiratory Medicine, Umeå University, SE-901 87, Umeå, Sweden; 2 Department of Applied Physics and Electronics, Thermochemical Energy Conversion Laboratory, Umeå University, Umeå, Sweden; 3 University/BHF Centre for Cardiovascular Science, University of Edinburgh, Edinburgh, United Kingdom; 4 Arrhenius Laboratory, Stockholm University, Stockholm, Sweden

**Keywords:** Biomass, Air pollution, Arterial stiffness, Blood pressure, Heart rate variability, Cardiovascular

## Abstract

**Background:**

Emissions from biomass combustion are a major source of indoor and outdoor air pollution, and are estimated to cause millions of premature deaths worldwide annually. Whilst adverse respiratory health effects of biomass exposure are well established, less is known about its effects on the cardiovascular system. In this study we assessed the effect of exposure to wood smoke on heart rate, blood pressure, central arterial stiffness and heart rate variability in otherwise healthy persons.

**Methods:**

Fourteen healthy non-smoking subjects participated in a randomized, double-blind crossover study. Subjects were exposed to dilute wood smoke (mean particle concentration of 314±38 μg/m^3^) or filtered air for three hours during intermittent exercise. Heart rate, blood pressure, central arterial stiffness and heart rate variability were measured at baseline and for one hour post-exposure.

**Results:**

Central arterial stiffness, measured as augmentation index, augmentation pressure and pulse wave velocity, was higher after wood smoke exposure as compared to filtered air (p < 0.01 for all), and heart rate was increased (p < 0.01) although there was no effect on blood pressure. Heart rate variability (SDNN, RMSSD and pNN50; p = 0.003, p < 0.001 and p < 0.001 respectively) was decreased one hour following exposure to wood smoke compared to filtered air.

**Conclusions:**

Acute exposure to wood smoke as a model of exposure to biomass combustion is associated with an immediate increase in central arterial stiffness and a simultaneous reduction in heart rate variability. As biomass is used for cooking and heating by a large fraction of the global population and is currently advocated as a sustainable alternative energy source, further studies are required to establish its likely impact on cardiovascular disease.

**Trial registration:**

ClinicalTrials.gov, NCT01488500

## Background

Exposure to fine and ultrafine combustion-derived particulate air pollution (PM_2.5_, particulate matter with a mean aerodynamic diameter <2.5 μm) is increasingly recognized as a short and long-term risk factor for cardiovascular disease and has been linked to the triggering of myocardial infarction within hours of exposure [1-5]. Controlled exposure studies have demonstrated that acute exposure to diesel exhaust and concentrated ambient particles, as models of urban particulate air pollution, generate acute vasoconstriction, increases in blood pressure and arterial stiffness [1,6,7], vascular endothelial dysfunction [8], myocardial ischemia [8,9] and changes in cardiac autonomic control [7,10]. This may in turn explain the observed increase in cardiovascular events [11].

Smoke from biomass combustion is the world’s oldest anthropogenic air pollution and contributes significantly to ambient levels of free radicals, polycyclic aromatic hydrocarbons, aldehydes, partially oxidised organic chemicals and particulate matter [12]. Currently more than half the world’s population relies on indoor burning of biomass for heating and cooking [13], and indoor exposure to PM_10_ (particulate matter with an aerodynamic diameter <10 μm) frequently exceeds concentrations of 1,000 μg/m^3^ in these settings [12]. This is to be compared to World Health Organization guidelines of annual average PM_10_ levels <20 μg/m^3^ and daily averages <50 μg/m^3^. If particulate air pollution from biomass smoke has similar effects on cardiovascular health as has been shown for other combustion derived air pollutants, this is of major importance for global public health; concerning low-income countries (where indoor open fires are commonplace) and high-income countries (where residential heating from wood stoves is popular) alike.

Although adverse effects on the respiratory system have been demonstrated in epidemiological as well as controlled exposure studies [12,14,15], less is known about the effects of biomass smoke on the cardiovascular system. Recent studies highlight associations between biomass air pollution and emergency department visits, hospital admissions for cardiovascular diseases [16,17] and cardiovascular mortality [18-20]. In an interventional study, indoor air pollution from biomass smoke was reduced significantly by replacing open fireplaces with chimney stoves and resulted in significant reductions in blood pressure [21] and myocardial ischemia measured using ambulatory electrocardiography [22].

In this study we tested the hypothesis that a 3-hour exposure to soot-rich wood smoke would cause acute haemodynamic effects evidenced by changes in heart rate, blood pressure, central arterial stiffness, and heart rate variability

## Results

Fourteen subjects participated in the study, and their baseline characteristics are reported in Table 1.

**Table 1:** Subject characteristics. Median values with inter quartile range or %

**Age, years**	26 (24-27)
**Sex**	Males: 8 (57%)
	Females: 6 (43%)
**Height, cm**	174 (166-182)
**Weight, kg**	74 (65-88)
**BMI, kg/m**^**2**^	24 (22-29)
**Vital capacity, L**	5.3 (4.3-6.5)
**Forced expiratory volume in 1 second, L**	4.0 (3.3-4.9)

**Figure 1:**
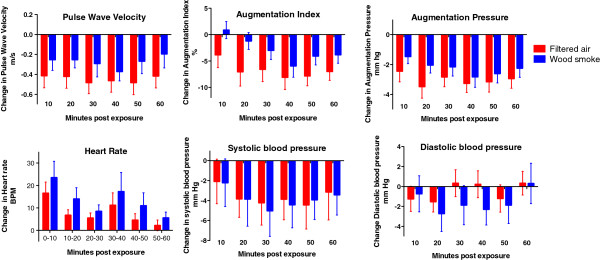
**Vascular parameters 10-60 minutes after exposure.** Compared to filtered air, wood smoke exposure increased heart rate (p = 0.008), pulse wave velocity (p = 0.005), augmentation index (p < 0.001) and augmentation pressure (p = 0.004), but no effect was seen on brachial systolic (p = 0.858) or diastolic (p = 0.213) blood pressure. All data expressed as mean ± standard error of the mean and P-values from 2-way ANOVA with repeated measures.

**Figure 2:**
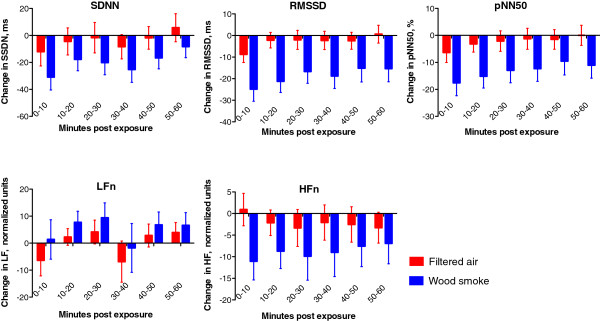
**Heart rate variability recordings in 10 min intervals 0-60 minutes post exposure.** Compared to filtered air, wood smoke exposure decreased the HF domain (p = 0.009), and a small decrease was seen in the LF domain (p = 0.086). All the time domain recordings were reduced following wood smoke exposure as compared to filtered air; SDNN (p = 0.003), RMSSD (p < 0.001) and pNN50 (p < 0.001). All data expressed as mean ± standard error of the mean and P-values from 2-way ANOVA with repeated measures.

At baseline there were no differences in heart rate, blood pressure, arterial stiffness or heart rate variability between exposure days (Table 2). Augmentation index, augmentation pressure and pulse wave velocity were all higher immediately after wood smoke exposure (p < 0.001, p = 0.004 and p = 0.005 respectively) as compared to filtered air (Figure 1). Wood smoke exposure did not affect systolic or diastolic blood pressure (p = 0.858 and p = 0.213 respectively), but did increase heart rate (p = 0.008) over the hour post exposure. Wood smoke exposure decreased general measures of heart rate variability (SDNN, RMSSD and pNN50; p = 0.003, p < 0.001 and p < 0.001 respectively) over the hour post exposure. Compared to filtered air, the high frequency domain (HFn) recordings were reduced (p = 0.009), consistent with vagal inhibition, whereas the low frequency domain (LFn) was marginally increased (p=0.086), although did not reach statistical significance Figure 2.

**Table 2:** Baseline haemodynamics and heart rate variability

	**Pre exposure filtered air**	**Pre exposure wood smoke**
**Systolic blood pressure, mmHg**	126.1±3.7	126.1±5.1
**Diastolic blood pressure, mmHg**	69.1±2.0	69.8±2.3
**Heart rate, beats per minute**	73.6±4.2	69.6±3.2
**Pulse wave velocity, m/s**	6.0±0.17	5.9±0.19
**Augmentation index, %**	−4.1±2.9	−7.0±2.6
**SDNN, ms**	73.9 (34.4-123.3)	87.7 (65.2-109.9)
**RMSSD, ms**	46.0 (20.6-65.1)	62.8 (35.9-80.5)
**PNN50, %**	23.7 (1.9-42.0)	35.0 (13.6-46.2)
**Triangular index, bin size 1:128**	14.4 (9.6-21.4)	19.9 (16.1-23.2)
**HFn, ms**	26.5 (14.3-40.6)	29.2 (13.2-50.6)
**LFn, ms**	72.5 (57.1-79.9)	69.2 (47.3-84.4)
**HF/LF, ratio**	0.37 (0.17-0.71)	0.43 (0.12-1.08)

Data expressed as mean ± SEM or median with inter quartile range. *SDNN* standard deviation of successive NN intervals, *RMSSD* root-mean square of successive NN interval differences; *HFn* high frequency components expressed as normalised units, *LFn* low frequency components expressed as normalised units, *HF/LF* high frequency/low frequency. There were no significant differences in any parameter pre exposure (p>0.05 for all). Values for heart rate, blood pressure and arterial stiffness from paired Student’s t-test and values for heart rate variability from Wilcoxon signed rank test.

## Discussion

In this study, a 3-hour exposure to wood smoke has been shown to simultaneously increase central arterial stiffness and heart rate and reduce heart rate variability, suggesting that wood smoke exposure does have acute haemodynamic effects on the cardiovascular system.

### Arterial stiffness

To our knowledge, this is the first study examining the acute effects of wood smoke exposure on central arterial stiffness. These results are consistent with previous studies reporting vascular impairment such as increased arterial stiffness after exposure to other air pollutants such as tobacco smoke (including second hand smoke) [23-26] and diesel exhaust [6]. In general, values reflecting arterial stiffness were low, indicating compliant arteries, and blood pressure was in the normal range, as expected in healthy young adults. As predicted, moderate exercise alone increased heart rate and decreased central arterial stiffness [27,28]. The exposure ends with a resting period within the chamber and the first measurements were taken 10 minutes post exposure. This allows 25 minutes to elapse between the last exercise and the first measurements of brachial blood pressure and arterial stiffness ensuring that the subjects are in a more relaxed state. As the exercise during both exposures was exactly the same, with only the nature of the exposure differing, we conclude that any differences observed in cardiovascular parameters would be a consequence of the wood smoke exposure.

Interestingly, both diesel exhaust and wood smoke appear to have similar acute and transient effects on central arterial stiffness. Following both pollutants, the increase in central arterial stiffness is more pronounced up to 20 minutes after the exposure and seems to decrease with time [6].

Epidemiological studies of exposure to particulate air pollution, however, only partially support a link between air pollution exposure and central arterial stiffness. A recent study including 3,996 subjects in six US cities failed to find an association between estimated average exposure to particulate matter air pollution over 20 years and arterial stiffness [29]. A study on patients that sought medical attention in hypertension outpatient clinics in Athens, Greece found a significant association between 5 day average PM_10_ and arterial stiffness in males [30]. These results do not necessarily contradict each other as long- and short-term modifiers of arterial stiffness differ. Long term modifiers include degenerative damage of elastin and an increase of collagen in the arterial wall, whereas short term modifications are more likely due to change in arterial tone and vascular endothelial signalling [31]. Arterial tone and vascular endothelial signalling may in turn be altered by factors affected by air pollution, such as the activity of the autonomic nervous system and vascular endothelial function [8,32].

### Heart rate variability

Variations in the interval between consecutive heartbeats are controlled by the autonomous nervous system and can be quantified by measuring heart rate variability. Decreased heart rate variability is a risk factor for cardiovascular morbidity and mortality in healthy individuals [33] as well those with existing cardiovascular disease [34,35]. In this study we have shown that general measures of heart rate variability are reduced following exposure to wood smoke, which appears predominantly due to a reduction in high frequency components of the spectrum suggesting vagal inhibition. In addition, our data show a small (but non-significant) increase in low frequency components, which may represent an increase in sympathetic activation. The alteration in the balance of autonomic innervation to the vasculature may explain the increased arterial stiffness and acute haemodynamic effects.

Wood smoke has been shown to rapidly trigger pulmonary vagal C-fibres in rats [36] and thereby activate the autonomic nervous system. However, in contrast to our findings, a recent controlled study on 10 healthy volunteers exposed to wood smoke failed to find evidence of an effect on the autonomic nervous system when assessed with heart rate variability [15]. Similarly, an intervention to reduce biomass smoke exposure of Guatemalan women showed reductions in ST-segment depression on electrocardiograms, but no evidence of alterations in heart rate variability [22].

There have been numerous studies addressing changes in heart rate variability following exposures to various air pollutants, including ozone, other gaseous pollutants, diesel exhaust and concentrated ambient particles. Results from these studies have often been contrasting and these inconsistencies may be due to differences in physicochemical properties of different pollutants as well as heterogeneity in study design. In a recent meta-analysis of 29 epidemiological studies comprising 18 667 subjects, Pieters and colleagues report an inverse relationship between heart rate variability in the frequency and time domains and exposure to PM air pollution [37]. Although ambient PM air pollution is derived from many different sources, our finding of a decrease in both the time and frequency domain following wood smoke exposure is in line with the epidemiological data reported by Pieters et al.

### Possible underlying mechanisms

Whilst it appears likely that the changes in central arterial stiffness are driven by an alteration in vascular autonomic control, vascular endothelial dysfunction, caused by reduced bioavailability of nitric oxide is a plausible alternative explanation. It is recognized that vascular function is intimately associated with the autonomic nervous system. Vascular dysfunction has been a consistent finding following exposure to concentrated ambient particles and diesel exhaust [8,38-40]. There have been some preliminary observations suggesting vascular endothelial dysfunction after exposure to wood smoke. One interventional study investigated the effects of HEPA filtration of subjects homes in an area with high levels of wood smoke derived air pollution and showed increased reactive hyperaemia following filtration of the air, suggesting that wood smoke does affect endothelial function [41]. However, a recent controlled wood smoke exposure study failed to show a significant effect on reactive hyperaemia in 20 healthy atopic subjects [42]. Though a credible mechanism, further research is necessary to establish whether endothelial dysfunction is driving the increase in arterial stiffness observed in this study.

The effects of air pollution on blood pressure has been heterogeneous regardless of study type and pollutant [1], and the fact that we did not see any effect on either systolic or diastolic blood pressure may have several explanations. First, our sample size may have been too small. Secondly, if vagal withdrawal increases heart rate, this may obscure an increase in blood pressure. Thirdly, arterial stiffness primarily reflect changes in central capacitance vessels, whereas brachial blood pressure is largely affected by peripheral resistance vessels [43], suggesting an effect of wood smoke on central rather than peripheral vessels.

### Study limitations

Biomass smoke is a complex mixture of particles and gaseous components with different physicochemical composition depending on source and combustion characteristics. The smoke from the partially air starved soot rich combustion used in this study may generate a different cardiovascular response as compared to a more efficient combustion in a modern wood stove or even poorer combustion during indoor cooking in low-income countries or combustion of other types of biomass.

Despite there being no significant differences in any cardiovascular measurement pre exposure, the results may have been influenced by regression to the mean as the results are expressed as change prom pre-exposure levels. The randomized double blind design of the study as well as a 20 min resting period prior to baseline measurements help mitigate this type of error.

## Conclusions

We have demonstrated for the first time that an acute exposure to wood smoke is associated with an increase in central arterial stiffness and heart rate along with a reduction of heart rate variability. These results imply that wood smoke exposure may be harmful to the cardiovascular system and that these effects are at least partially mediated by the autonomous nervous system. Considering the global prevalence of wood smoke, this calls for further studies investigating wood smoke exposure as a possible risk factor for the development of cardiovascular disease as well as mechanistic studies elucidating possible underlying pathophysiological mechanisms.

## Methods

Fourteen non-smoking volunteers completed the study. The subjects underwent a basic screening medical examination and were all healthy with normal electrocardiogram, respiratory function and blood chemistry. None reported any symptoms of respiratory illness at least 6 weeks before or during the study. The study was approved by the local Ethics Review Board, performed in accordance to the Declaration of Helsinki and with the written informed consent of all participants.

### Study design

The subjects attended on two separate occasions at least three weeks apart and were exposed to filtered air and diluted wood smoke (target PM_1_ concentration 300 μg/m^3^) for 3 hours in a randomized double-blind crossover fashion. During the exposure, the subjects performed intermittent exercise on a bicycle ergometer alternated with rest at 15-minute intervals to achieve an average minute ventilation of 20 L/min/m^2^. The subjects abstained from alcohol and caffeine 24 hours before the exposure.

### Wood smoke exposure

Wood smoke was generated using a common Nordic wood stove (chimney stove) in a controlled incomplete combustion firing procedure. Birch wood logs with a moisture content of 16-18% were inserted every 5-15 minutes to maintain a high burn rate with repeated air-starved conditions. Accordingly, excess oxygen in the flue gases varied from 3 to 17%. Due to the varying combustion conditions, CO concentrations in the flue gases varied from a typical range of 1000-5000 ppm to peaks up to around 12 000 ppm during low excess oxygen (<5%) conditions. In previous studies it has been illustrated that this kind of combustion with high burn rate of wood is associated with high elemental carbon (EC), soot and polycyclic aromatic hydrocarbon (PAH) emissions [44].

The wood smoke was diluted with HEPA and activated carbon filtered air in three steps and continuously fed into a controlled environment exposure chamber (15.3 m^3^) to achieve a steady state concentration. The atmosphere in the chamber was monitored for gaseous pollutants using continuous measurement of nitrogen oxides (NO_x_) (chemiluminescence, CLD 700 Ecophysics, >0.001 ppm) and carbon monoxide (CO) (IR, UNOR6N Maihak). PM_1_ (particulate matter with an aerodynamic diameter of <1 μm) mass concentration was measured on-line using a tapered element oscillating microbalance (TEOM 1400 by Thermo Scientific) equipped with a PM_1_ pre-cyclone. Integrated with the TEOM, a filter (Teflon) sampling line was used to determine the particle mass concentration gravimetrically. Within the exposure chamber the diluted wood smoke had an average PM_1_ concentration (measured with the TEOM) of 314 μg/m^3^ (range 232 - 356 μg/m^3^), which was associated with average NOx and CO concentrations of 0.41 ppm and 25 ppm, respectively. Due to the combustion procedure, as described above, the concentration of PM and gases in the chamber did vary, although the aerosol residence time in the chamber (~20 min) compensated for this.

The aerodynamic diameter of the wood smoke particles was measured in the aerosol flow into the chamber using a scanning mobility particle sizer (SMPS). The SMPS was used for regulating the dilution during exposures to reduce fluctuations in the chamber. Due to the placement of the SMPS, data on particle size and number concentration in the breathing zone of the subjects is not available. At the inlet, the size distribution was bimodal with one peak at around 50 nm and a second peak between 100-200 nm. Previous studies show that the 50 nm peak consist of alkali salt particles (e.g. K_2_SO_4_ and KCl) and the 100-200 nm peak consist of a soot mode with more or less organic material [45,46]. Organic (OC) and elemental carbon (EC) was determined using a thermal-optical carbon analysis (Method NIOSH 5040) [47]. Polycyclic aromatic hydrocarbons (PAH) were sampled in the chamber by glass fibre filters (Ø 47 mm) for the particulate fraction followed by a polyurethane foam (PUF) plug (Ø 75 mm × 50 mm) for the semi-volatile fraction. PAH compounds (3-6 rings) were analyzed by gas chromatography-mass spectrometry (GC-MS) as described elsewhere [48,49].

The total carbonaceous (TC) PM of the wood smoke was dominated by EC with a EC/TC ratio of 0.72 ± 0.08. Based on estimation of the components of the total PM in the wood smoke, the PM consisted of 38% soot, 24% organics and the remainder presumably alkali salts. A factor of 1.8 was used to convert the OC content to total organic PM and a factor of 1.1 was used to convert EC to soot PM mass concentration.

The total PAH concentration in the chamber was 1.1 ± 0.7 μg/m^3^, of which 74% (0.78 μg/m^3^) was in the particulate phase. The 12 dominating PAH compounds in the PM fraction, accounting for 86±2% of the total analyzed PAH, were (in descending order); Benzo(a)pyrene, Benzo(b)fluoranthene, Benzo(ghi)perylene, Benzo(e)pyrene, Benz(a)anthracene, Indeno(1,2,3-cd)pyrene, Benzo(k)fluoranthene, Benzo(ghi)fluoranthene, Coronene, Pyrene, Perylene and Fluoranthene Table 3.

**Table 3:** Wood smoke exposure characteristics

	**Unit**	**Average**	**Stdev**	**Max**	**Min**	Exposure variation^*^
PM_1_ mass conc. (TEOM)	μg/m^3^	314	38^**^	356^***^	232^***^	200-400
PM_1_ mass conc. (filter)	μg/m^3^	294	36	351	246	
CO	ppm	25	6	35^***^	16^***^	10-40
NO_x_	ppm	0.41	0.12	0.65^***^	0.26^***^	0.2-0.7
EC/TC (elemental/total carbon)		0.72	0.08	0.84	0.57	
Organic fraction of total PM_1_^****^	%	24	8.0	40	16	
Soot fraction of total PM_1_^*****^	%	38	9.9	53	28	
PAH - PM associated (from filter)	μg/m^3^	0.78	0.56	1.94	0.18	
PAH - semi-volatile (from PUF)	μg/m^3^	0.28	0.12	0.53	0.14	

^*^ Typical variation interval during an exposure for the continuous measurements.

^**^ Standard deviation of the average TEOM data from each exposure.

^***^ Max and min of the average from each exposure.

^****^ Estimated based on the OC analysis and TEOM (assuming OC*1.8 in total organic PM weight).

^*****^ Estimated based on the EC analysis and TEOM (assuming EC*1.1 in total soot PM weight).

### Arterial stiffness and blood pressure

Vascular studies were performed in a quiet, temperature-controlled room with the subjects resting in a semi-recumbent position. Measurements were performed at baseline (pre exposure), and at 10-minute intervals after the exposure for one hour.

Blood pressure was measured using a validated semi-automated oscillometric sphygmomanometer (Boso-Medicus, Boso, Jungingen, Germany). Central arterial stiffness measured by pulse wave analysis was determined with a high-fidelity handheld tonometer (Millar Instruments, Texas, USA) at the right radial artery using the SphygmoCor™ system (AtCor Medical, Sydney, Australia) in accordance with the manufacturer’s recommendations. Briefly, pulse wave analysis derives an aortic pulse wave from a recording at the radial artery and the brachial blood pressure via generalized transfer function [50]. From this, augmentation index and augmentation pressure, both estimates of central arterial stiffness, are calculated. As augmentation index decreases with increased pulse rate due to a shorter ejection duration of the left ventricle [51], all measurements are corrected for pulse rate.

Carotid-femoral pulse wave velocity is regarded as the current “gold-standard” measurement of central arterial stiffness due to its reproducibility, ease of use and ability to predict cardiovascular events in several large trials [43,52,53]. Pulse wave velocity measurements were made using the Vicorder system (Skidmore Medical, UK), which detects the pressure wave simultaneously at the carotid and femoral arteries using inflatable cuffs. The pulse transit time between these sites is calculated by identifying the arrival of the foot of the pulse wave using a proprietary intersecting tangents algorithm as compared to the R-wave on the electrocardiogram. The distance between the sites divided by pulse transit time is then used to generate pulse wave velocity.

### Heart rate variability

Continuous 3-lead electrocardiographic recordings were obtained through the study period using a Holter monitor (Spacelabs 90217, Spacelabs, UK). Heart rate variability and heart rate was determined from the electrocardiographic recordings using the Reynolds Medical Pathfinder Digital 700 Series Analysis System (Delmar Reynolds, UK) and the HRV Tools software package (Delmar Reynolds, UK) pre exposure and at 10-minute intervals over the hour post exposure. Standard time domain measures were calculated including the mean NN interval (time interval between consecutive sinus beats), SD of NN interval values (SDNN), percentage successive NN interval differences >50 ms (pNN50), root mean square of successive NN interval differences (RMSSD) and the triangular index. Frequency domain analysis included the low frequency (LF) and high frequency (HF) components of the power spectrum. LF and HF and are expressed in normalised units (LFn and HFn), to account for variation in the total power as well as the HF/LF ratio. No geometrical methods are reported for the 10 minute intervals over the hour post exposure due to the difficulties obtaining accurate measurements of these indices from short term recordings [54].

### Statistical analysis

All data was analysed using GraphPad Prism, version 5.0, (GraphPad Software, USA) by 2-tailed paired Student’s t-test, Wilcoxon signed rank test and 2 way analysis of variance (ANOVA) with repeated measures where appropriate. Statistical significance was taken at P < 0.05. Data are presented as mean ± standard error of the mean (SEM) or median with inter quartile range. All data analysis was made prior to unbinding the nature of the exposures.

To avoid bias from day to day variations in blood pressure, arterial stiffness and heart rate variability, all measurements over the hour post exposure are expressed as change from baseline taken prior to each exposure. Post exposure values were all normally distributed (D’Agostino-Pearson normality test).

## Abbreviations

PM: Particulate matter; PM1: Particulate matter with an aerodynamic diameter <1 μm; PM2.5: Particulate matter with an aerodynamic diameter <2.5 μm; OC: Organic carbon; EC: Elemental carbon; PAH: Polyaromatic hydrocarbons; SMPS: Scanning mobility particle sizer; TEOM: Tapered element oscillating microbalance; NN: Interval between consecutive heart beats; SDNN: Standard deviation of the NN interval; RMSSD: The square root of the mean squared differences of successive NN intervals; pNN50: Percentage of successive NN intervals greater than 50 ms; HF: High frequency component of heart rate variability; LF: Low frequency component of heart rate variability.

## Competing interests

The authors declare no competing interests.

## Authors’ contributions

AB, TS, NM, DN, JL and JB conceived and participated in the design of the study. JU, AM and JB carried out the clinical studies and collected the data. JU, JB and JL performed the data analysis and statistical analysis. CB and RN generated the clinical exposures and performed analysis of the exposure variables along with RW. JU drafted the manuscript and all authors were involved in critical review. All authors read and approved the final manuscript.

## Support

Swedish Heart Lung Foundation, Swedish Clean Air Research Program, British Heart Foundation, Umeå University, Västerbotten County Council
